# Beliefs around help-Seeking and Support for Dementia in the Australian Arabic Speaking Community

**DOI:** 10.1177/14713012231166170

**Published:** 2023-03-29

**Authors:** Issra Allam, Meredith Gresham, Lyn Phillipson, Henry Brodaty, Lee-Fay Low

**Affiliations:** 522555 The University of Sydney Faculty of Medicine and Health, Sydney, NSW, AU; Centre for Healthy Brain Ageing, 98994 University of New South Wales Faculty of Medicine, Sydney, NSW, AU; Australian Health Services Research Institute, University of Wollongong, Wollongong, NSW, AU; Centre for Healthy Brain Ageing, University of New South Wales Faculty of Medicine, Sydney, NSW, AU; The University of Sydney Faculty of Medicine and Health, Sydney, NSW, AU

**Keywords:** dementia, alzheimer’s, carers, Arabic community, help-seeking, ethnic minority

## Abstract

**Background:**

The number of people with dementia in multicultural Australia is rapidly increasing. Despite its culturally diverse population, there is limited research about how people from ethnic minority groups understand and approach help-seeking and support for dementia. The aim of this study is to understand the perceptions of dementia symptoms, help-seeking and support in the Australian Arabic-speaking community.

**Methods:**

This study used a cross-sectional qualitative research design. Individual, semi-structured interviews using projective stimulus techniques were used. Participants were three Arabic-speaking people aged over 70 who were experiencing cognitive changes or dementia symptoms, six carers, and five health or social care practitioners experienced in working with Arab-Australians. Phone or video chat interviews were conducted in either Arabic or English. Interviews were audiotaped, translated when needed, transcribed verbatim and inductive thematic analysis was undertaken.

**Findings:**

Seven *themes* were identified. Participants described dementia as relating to symptoms of confusion and memory loss. Carers and older people believe that when older people are experiencing these cognitive symptoms, they must be cared for primarily by ensuring their happiness and comfort. Barriers to help-seeking and support included a lack of help-seeking due to cultural norms of family orientated care, families are unsure of where to seek help and fear of community judgement. Two ways to facilitate help-seeking and support were to build trust through culturally appropriate support and to educate the community.

**Conclusion:**

Family, trust and community were identified as central pillars of the Australian-Arabic-speaking community. There is a need to increase dementia literacy in this community particularly around help-seeking and decreasing stigma. Education should be promoted by trusted community members and religious leaders. As the first point of professional contact, general practitioners need to be upskilled to support Arabic-speaking Australians around dementia.

## Introduction

Internationally, societies are increasingly multicultural. In 2019 it was estimated that 3.5% of the global population (272 million) migrated from their country of origin, with around 13.5% of these people being over 65 years of age (Alladi & Hachinski, 2018; International Organization for Migration, 2020; United Nations, 2016). Health issues for older migrant populations including dementia, have not been a focus of research and policy, possibly due to assumptions that the issue of dementia is marginal or that most first world nations receiving migrants have sufficient mainstream support to cater for needs (Livingston et al., 2020; Truswell, 2016). However cultural diversity presents challenges for healthcare systems to deliver culturally appropriate care (Sagbakken et al., 2018). Ethnic communities in Canada, USA, UK, and Australia have been found to underuse dementia services; older migrants with dementia are diagnosed later in the disease, and if diagnosis is made, are less likely to be prescribed medication, participate in research, or receive appropriate care (Sagbakken et al., 2018; International Organization for Migration and League of Arab States, 2004).

In Australia, 25% of the population is born overseas and it is estimated that one-in-five people living with dementia are from ethnic minority backgrounds. The Arabic-speaking community constitutes around 1.4% of the Australian population, and Arabic is the second most spoken non-English language (Australian Bureau of Statistics, 2015). Arabic-Australians are from diverse countries (e.g., Iraq, Lebanon, Syria, and Egypt), with diverse religions (including Islam, Christianity, and Judaism), ethnicities (including Persians, Kurds, Berbers, and Jews) and are of diverse socioeconomic and educational backgrounds (Harb, 2016).

Despite diversity, the Australian Arabic-speaking community commonly adopt a filial piety view of caregiving for older family members as a normal part of life (Bergeron & Lagacé, 2021; Jutlla, 2013; Mukadam et al., 2011). Seeking help from external services is seen as disrespectful and a failure to fulfil family obligations (Brijnath et al., 2021). Social care institutions and nursing homes are often perceived by Arab communities as elderly abandonment, and the stigma associated with care or support outside of the family unit is a significant barrier in seeking help (Shanley et al., 2012; Aryal, 2017). The belief that cognitive change is a normal part of ageing, as well as the lack of knowledge of the role of specialist services, frequently results in delay of Arabic communities seeking diagnosis or assistance, and general practitioners may only be consulted when dementia symptoms are pronounced (Karam & Itani, 2013; Daher-Nashif et al., 2021). Perceptions of dementia in Lebanese Arabs is commonly negative. A survey of 254 Lebanese adults revealed that people with dementia were seen as dependent and helpless, and provoked reactions of fear or even anger (Hamieh et al., 2019). Furthermore, dementia may be understood in Islamic-Arab communities as a mental illness and may be interpreted as a divine test or punishment, and assistance is more likely to be sought from a religious leader than a health professional (Abbasi & Paulsen, 2019). In Arabic, a common word used for dementia is *kharaf,* which describes ‘unravelling’ or ‘losing one’s mind’ (Daher-Nashif et al., 2021). This conflation of dementia with mental illness compounds reluctance to openly talk about dementia. Help seeking is further hampered by the desire to keep care within the family unit, a lack of knowledge about services, as well as cultural incompatibility of services (Alzheimer’s Disease International, 2019). Negative stereotypes and stigma concerning dementia may affect the way in which Arab-Australians describe dementia, distorting communication between families and health professionals, and contributing to failure to meet needs (Saleh et al., 2012). Some health professionals may not understand cultural perceptions of dementia, inadvertently disadvantaging access of minority ethnic groups to appropriate support (Atkin, 2010). Perceptions of dementia are influenced by a person’s education levels, family background, economic circumstances, and their migration experience however, without language-specific supports, English-language proficiency and cultural understanding, people from the Arabic-speaking communities may experience challenges to enter and navigate the Australian age care system (Australian Medical Association, 2020; El Masri et al., 2017; Healthdirect, 2018; Nielsen et al., 2021).

Globally, there is a paucity of literature that explores dementia care in ethnic and migrant communities, including the Arabic-speaking community in Australia (Al Abed et al., 2014; Truswell, 2016). This study aims to explore the beliefs around help-seeking and support for dementia in the Australian Arabic-Speaking community.

## Methods

### Research Design and Questions

A cross-sectional qualitative research design was used to obtain a snapshot of beliefs around help-seeking and support regarding dementia within the Australian Arabic-speaking community in metropolitan Sydney, Australia (Godwill, 2015; Mann, 2003; Setia, 2016).

Research questions are:

• How do Arab-Australians understand and respond to symptoms of dementia?
• What are the barriers and facilitators to help-seeking and support for dementia?

### Ethics, Recruitment, and Participants

Approval was obtained from UNSW (Sydney) Human Ethics (#HC190776). Written, informed consent was obtained from all participants prior to their participation.

Using purposive sampling, recruitment was conducted by the bilingual first author (IA) through Arab-Australian community groups, residential aged care facilities, community-based services, and personal networks. Purposive sampling was used to achieve variation in age and health professional background or experience to support a range of views (Etikan et al., 2016). The three participant categories were, (1) Arabic-speaking people over the age of 70 who are experiencing self- or family-reported dementia-like symptoms, (2) carers who support older Arabic-speaking people with a range of diagnoses, including dementia, respiratory disease, diabetes, kidney failure, dementia-like symptoms without a formal diagnosis, and (3) health and social care practitioners (HSCPs) working with older Arabic-speakers and their carers. Inclusion criteria for participation was provision of informed consent, being over 18 years, and ability to communicate verbally in English or Arabic. Triangulation of perspectives from carers, older people and HSCPs was essential to achieve a broad understanding of views where minimal research has been conducted.

## Data Collection

Individual, semi-structured interviews were conducted by the first author. Due to COVID-19 regulations preventing face-to-face contact, interviews were conducted using video conferencing or telephone. Demographic information including age, gender and diagnoses were collected prior to the interview.

Interview questions were tailored for each participant group, and projective techniques were used (Phillipson et al., 2022). Projective techniques are those which use indirect ways of eliciting feelings, attitudes, and beliefs about situations. In this study, participants were shown a series of photographs of a hypothetical older couple, called Salma and Adel, and were asked to reflect on thoughts and feelings that this couple may be experiencing, and the nature of actions they may take as they are confronted by Salma’s dementia-like symptoms.

The process for older people with dementia-like symptoms was: The first photo ‘introduced’ participants to the couple and gave brief information about symptoms Salma has been experiencing was given. These were feeling low, difficulty in concentrating, difficulty in making a meal, and being confused about where she is. The participant was asked what came to mind when hearing about these symptoms. A second photo showed Salma sitting alone, in a thoughtful pose and the participant was asked what Salma was thinking, feeling and what she might do in response to these symptoms. A third photo showed the couple at a doctor’s surgery where the doctor is delivering the diagnosis, “Salma, you have dementia”. Again, the participant was asked what Salma might be thinking and feeling. The same process was followed for the carer, but with photos of the hypothetical carer, and the carer participants were asked to reflect on what the thoughts, feeling and actions of the hypothetical carer might be. The interviewer did not use the word ‘dementia’ until late in the interview, as stigma associated with the condition may have influenced discussion. (Connelly & Peltzer, 2016; Grossoehme & Lipstein, 2016). Photographs used were specifically developed to be culturally appropriate for the intended audience. Personal associates of the first author (IA) gave written consent to be photographed and their images used in this study. Older person and carer projective technique stimulus material are at supplemental material Appendix A and B.

For HSCPs, a projective technique involving a free association exercise using the words ‘dementia’ and ‘support’ was used. Participants were asked to say what came to mind, including thoughts, feeling, and attitudes when seeing these words. HSCP participants were asked open-ended questions about their experience with older Arab-Australian clients and carers, their opinions about barriers and facilitators this group may have in help seeking and the nature of current post diagnostic support for this group. HSCP projective technique stimulus materials are at supplemental material Appendix C.

All interviews were audio recorded, translated as required and transcribed verbatim. NVivo (QSR International, 2020) software was used to analyse and store data.

### Data Analysis

Data was thematically analysed. Research questions were used to guide the inductive approach to capture and explore relevant themes (Braun & Clarke, 2006). Data was coded, and main code patterns combined and categorised into themes by IA and LFL. Where discrepancies arose, themes were discussed until consensus was reached.

Reflexivity was identified as a potential factor that may distort data analysis due to IA’s Arab cultural background. Cultural assumptions and values have the potential to influence analytic processes (Korstjensa & Moser, 2018). During supervision, a process of conscious review between IA, LFL and MG was undertaken to maximise the validity of the findings (Bishop & Shepherd, 2011).

## Findings

### Participant Characteristics

14 interviews ranging in duration between 14 and 51 min were conducted (Table 1).

**Table 1.** Participant Role, Gender, and Age.

	Male	Female	Age in years, mean (range)	Total
Older person	1	2	67 (55–78)	3
Carer	0	6	48 (32–64)	6
HSCP	1	4	41 (21–61)	5
Totals	2	12	--	14

All participants were from Sydney, New South Wales, Australia. HSCPs worked in a variety of sectors including health, public and private aged care, community care and National Disability Insurance Scheme (NDIS). Work roles were assistant-in-nursing, disability services provider, nursing home general manager, multicultural access project officer, and dementia care navigator.

### Key themes

Seven themes were identified that mapped to the three research questions. These are summarised in Table 2.

**Table 2.** Summary of research themes.

Research questions	Associated themes
How do Arab Australians understand and respond to symptoms of dementia?	• Memory loss and confusion, resulting in childlike behaviours
	• Make them happy and comfortable through supporting routine and everyday activities
What are barriers to help-seeking and support?	• If someone is sick, it’s something to be hidden • Lack of help-seeking due to cultural norms of family-orientated care • Families are not sure where to go for help
What are facilitators of help-seeking and support?	• Building trust through culturally appropriate support • Education of the community

### How do Arab Australians Understand and Respond to Symptoms of Dementia?

#### Memory Loss and Confusion

Older people and carers viewed dementia as a disease that primarily involved confusion and memory loss, with most participants stating that this led to loss of ability of the older person to look after themselves. Examples given included memory loss leading to forgetting to eat, and confusion resulting in wandering and becoming lost. Some carers spoke about the person living with dementia forgetting their children. These symptoms were also generally associated with mental illness.

“Not only did she forget everybody, [but] she [also] forgot everything.” (Female, carer, age 40)

Carers differentiated ‘normal’ forgetting with the forgetfulness experienced by people living with dementia, which impacts the ability to interact with others and perform daily activities. Examples given included putting the milk in the cupboard instead of the fridge, and not being able to cook as well as previously. These changes were associated with the loss of one’s identity and becoming child-like, and would evoke emotional responses of pity, sadness or distress in the carer.

“Their mind is like the mind of a little child. A baby.” (Female, carer, age 64)

The analogy of ‘child-like’ behaviours was extended to symptoms of socially inappropriate behaviours. Participants described ‘tantrums’, inappropriate undressing and increasing dependency in self-care tasks, such as dressing and bathing.

#### Make them Happy and Comfortable through Supporting Routine and Everyday Activities

Carers expressed the main goal of care for older people, including those diagnosed with dementia, was to ensure that they are comfortable and relaxed, characterised as surrounding the older person with family and friends, buying them new clothes, and taking them on outings.

“You feel like you want to do everything in your power to make them not feel low… to be happy again…” (female, carer, age 34)

Carers and older people emphasised the importance of keeping a routine and helping the person with dementia to continue looking after themselves and doing everyday activities around the home such as cooking and cleaning, to assist in preserving a sense of normality and stability as their functional ability changes. Establishing a routine was viewed as a way of achieving a sense of mastery over their daily life and minimising distress.

“At home… do the normal things that she always used to do so she doesn't think of it [the dementia symptoms]” (female, carer, age 35)

Activity limitations were recognised, and interviewees provided the caveat that people with dementia should not be ‘pushed’ to do too much. Instead, carers should work alongside the person with dementia in partnership.

“It is not important that she completes everything in one day… it shouldn’t bother her or make her feel like she did something incomplete.” (Male, older person, age 78)

### What are the Barriers to Help-Seeking and Support?

#### If Someone is Sick, it’s Something to be Hidden

Both carers and HSCPs reported Arabic-speakers experience stigma and self-stigma around illness, and spoke of feelings of fear, shame, and embarrassment about what the community would think of them if experiencing symptoms of memory loss, confusion, or disorientation. Older people and families were reluctant to talk about their symptoms and tried to actively hide them.

“They will try not to talk about what is happening to them so that the people around them won’t say things like ‘this person’s mind is small.” (Female, carer, age 64)

Older people spoke of becoming defensive if their symptoms were mentioned. One carer said that their family do not mention their mother’s memory loss in front of her, as talking about it may lead to a “*psychological crisis*.” Instead of reaching out for help, the person experiencing dementia symptoms will try to continue to carry out their normal activities of daily living and attempt to hide their symptoms. In this way, they “*try to give you a better picture that they are coping*”.

“… I think it's a bit of a taboo topic… there's not many people who like to verbalise what's happening in their homes” (Female, assistant-in-nursing, age 21)

HSCPs spoke of strong fear of judgement about dementia-like symptoms within the Arabic community. In contrast to carers’ catering to the happiness of older people by *“taking them out, buying them new clothes”*, health professionals described families keeping older people at home to avoid social embarrassment should the older person have ‘inappropriate’ behaviours.

“Arabic culture sometimes, they do hide when someone has dementia. They don't go out themselves, they keep them inside and indoors and they don't understand what to do.” (Female, nursing home manager, age 35)

#### Cultural Norms of Family-Orientated Care means that we do not Seek Outside Help

All participant groups emphasised Arabic cultural values of family-oriented care and support. Participants expressed that caring for parents is an obligation that should be embraced to “*pay them back*” for their sacrifices.

“They [the children] do want to help their parents. So, there is that delegation of responsibility.” (Female, assistant-in-nursing, age 21)

Despite care of older people being viewed as a cultural obligation, carers described the role as an important responsibility that is also their source of happiness.

“I feel like I am doing something good. I am doing this to help him to make him happy and that he is not upset with his life.” (Female, carer, age 64)

While caring was seen as the woman’s role within the family, close and extended family members all contributed to ensure that the older person is well supported. Participants expressed that this shared the load, reduced stress and allowed adult children to manage work, home and childcare responsibilities while caring for their older parents. It was noted that keeping care within the family unit may stem from habits of family-reliance after migrating to Australia and beliefs that care outside the family unit would be sub-standard.

“Arab community tend to be very… self-sufficient… bringing support care worker into the house or sending the person to a nursing home (is) almost as if they failed.” (Female, assistant-in-nursing, age 21)

“Because the nursing home does not provide the compassion and caring nature that [the older person] would receive from the children…” (Male, older person, age 78)

Older people and carers described adult children placing parents in nursing homes as “*heartbreaking”* and disrespectful. If older people are admitted to a nursing home due to complex medical issues, HSCPs stated that families still involve themselves in daily care routines of the older person including dressing and feeding. While caring for an older person is associated with gratefulness and satisfaction at being able to fulfil family obligations, participants spoke of the stress of providing care indicating that dementia affects not only the person who has been diagnosed, but their family support networks, and these changes in family dynamics require patience. The children of the older person may become overwhelmed by their caring responsibilities. HSCPs observed that older people and their families struggle without asking for external support, since seeking external help was perceived to be a failure to carry out one’s family duties.

“And when they approached us [age-care providers] and they can seek for help because they’re struggling when they are coming towards the end of dementia.” (Female, nursing home manager, age 35)

Older people born overseas were less willing to accept services and external help, however the next generation took more initiative to “*read up about it [dementia]*.” One HSCP stated that the younger generation understands that some conditions require higher levels of care than family can provide and gave examples of complex medication administration, management of blood pressure and oxygen levels.

“The mindset [of the Arabic-speaking community] is slowly shifting where they are more willing to seek help… We can ask for help and advice more than our parents did when they were struggling.” (Female, carer, age 32)

#### Families are not sure where to go for help

HSCPs expressed that carers in the Arabic-speaking community “*don’t know what’s out there to actually even seek that help.”* The Australian aged care system is viewed as complex and both carers and HSCPs spoke of the lack of accessible information about diagnosis, services, and support. Moreover, the sense that dementia has no treatment led to a sense of futility in seeking information or support:

“Like I'm trying to find information. If there's no treatment, what's the point of knowing? Like having it officially told or diagnosed” (Female, carer, age 40)

### What are the Facilitators to Help-Seeking and Support?

#### Build Trust Through Culturally Appropriate Support

Trust was a central theme for all participants. A prerequisite for the Arabic-speaking community to seek help or accept services was trust in those delivering services and a perception that the service is respectful and competent. Older people and carers were fearful of mistreatment by services, particularly after hearing about negative incidents from friends or media.

“… It doesn’t matter if their Arab, or English or French or Turkish… the important thing is that this place helps people in the respectable way with good intentions…” (Male, older person, age 78)

If a trusting relationship with services was not established, HSCPs described families as not willing to disclose the severity of their situation and will continue to struggle in silence.

“You really need that trust because otherwise you're just going to paint a good picture and kind of request services that you really don't need or it's not very applicable to the situation and you just cope with it.” (Female, multicultural access project officer, age 35)

Culturally appropriate care was seen as fundamental by HSCPs for provision of support to the Arabic-speaking community. HSCPs explained that Arabic-speakers have different needs from other ethnic minority communities, including preference for same-gendered care, cultural foods, and religious interventions, especially when explaining dementia. Support from HSCP was desired through a family-centered approach rather than focusing on the individual, which is congruent with community values. Carers thought that service providers who understand their language and culture, provide cultural food, and understand the person’s expressions of pain and needs are central to quality care.

“It's not just about having leaflets in Arabic… so culturally appropriate it doesn't mean just language or understanding culture.” (Male, age-care consultant with experience in both community and residential age care sector, age 38)

#### Active Outreach for the Community

All participant groups expressed the need for raising awareness and education. For families, there was the need to understand that dementia was not ‘just old age’ as well as where to go for help and how to support a person with dementia. HSCPs expressed that education needs to come from a trusted and respected authority in the community, recommending religious leaders provide education about accepting help from services and nursing homes, and recommending general practitioners provide education about symptoms and management. GPs were also seen to have a role in linking families to appropriate services and explaining to them the situation of their loved ones.

Older people and carers believe that community organisations must actively intervene with struggling families. They expressed families may be unaware of the skills needed to be able to care for a person living with dementia. Older people and carers wanted education about dementia in a “staged approach”. This enables families to have time to understand and accept their changing circumstances.

“But you've got to give people time to understand it and accept it. And you storming into someone who just had a diagnosis, really overwhelmed with what the future's going to look like.” (Female, HSCP with experience with carers from culturally and linguistically diverse backgrounds who support people with dementia, age 61)

## Discussion

This study aimed to explore beliefs and help-seeking around dementia in the Arabic-Australian community. Findings indicate that Arabic-speaking Australians understand dementia as confusion and memory loss in older age, but due to community stigma around dementia, sickness and mental illness specifically, symptoms of dementia are often hidden by the person and their family. Arab-Australians in this study expressed that a strong sense of familial duty to maintain care within the family was a barrier to help-seeking, and that building trust and community education through respected community leaders would be a major factor to facilitate accepting help-seeking and support outside the family unit.

We found that some participants conflated dementia with mental illness, while most described people with dementia as having reduced self-efficacy, behavioural and emotional disturbance. Arabic-speaking communities living in both Western and Arab countries, stigmatise mental illness (Zolezzi et al., 2018). It appeared that stigma underpins families’ desire to try to hide dementia symptoms due to fear of shame and negative community judgement. While stigma about mental illness is common in many societies, the collective nature of the Arabic-speaking community might heighten the impact of stigma (Alhomaizi et al., 2018) including associative stigma, where the family of the person with dementia are afraid of being shamed and judged. It was clear that older people and their carer would delay or avoid help-seeking for dementia due to stigma.

While supporting someone with dementia symptoms was mainly kept within the family unit, our findings suggest that GPs were a central point of professional contact when families notice dementia symptoms from an older person. This is similar to the help-seeking practices of migrants from other backgrounds (Brijnath et al., 2021; Robotin et al., 2021; Zolezzi et al., 2018). However, GPs can be reluctant to formally diagnose dementia if the person does not want the diagnosis (Phillips et al., 2012). Therefore, GPs should be supported when speaking to Arabic-speaking patients about the possibility of dementia to avoid delayed diagnosis and promote early interventions.

Consistent with studies of other migrant communities, we found that Arabic-speakers will accept help from trusted services (Colagiuri et al., 2007; Saleh et al., 2012; Shanley et al., 2012). Nursing home placement was found in our study to be seen as heartbreaking and disrespectful to the older person. However second-generation participants in our study emphasised the importance of community services in supporting the person living with dementia and their carers. This contrasts with an Australian multicultural study that found that in-home care services were considered incompatible with their values of filial piety (Shanley et al., 2012).

Key strengths of this study included a bilingual researcher with personal understanding of the Arabic-speaking community and its cultural norms, and the use of culturally appropriate study materials in Arabic and English. This minimised language barriers to research participation (Shanley et al., 2013). The use of non-direct questioning through projective techniques supported conversations around dementia and may have assisted older participants with memory deficits feel more comfortable by not having to remember specific information about their own experiences. Limitations were the small sample size due to recruitment difficulties, caused in part by COVID-19. While the final few interviews did not produce new themes (Saunders et al., 2018), a larger sample would have confirmed that saturation was reached.

There was little diversity in nationalities of participants, with most identifying as having a Lebanese heritage (10 out of 14 participants) and 12 of out 14 participants being Muslim. Lebanese are the most prevalent Arabic-speaking community in Australia, representing 64% of Arabic-speakers (Mazbouh-Moussa & Ohtsuka, 2017). People from other nationalities (i.e., Egyptian, Syrian, or Iraqi) and religions (i.e., Islam, Christianity, or Judaism) may have different attitudes, needs and behaviours (Kenny et al., 2005). The findings may be influenced by self-selection bias. Participants with more exposure to dementia or with lower dementia stigma may be more willing to disclose their experiences, compared to the broader Arabic-speaking community. Therefore, our findings may be more positive than other attitudes regarding help-seeking and support in this community.

Further research might include a quantitative approach (i.e., community survey) building on the findings of our study to explore how common our findings are. Intervention research could co-design and evaluate community programs to increase dementia literacy and help-seeking in Arabic communities. The projective stimulus techniques used here could also be employed with other CALD communities to explore other culturally sensitive topics.

Our findings suggest that more education about dementia from community leaders is required to address misconceptions and facilitate help-seeking in the Arabic-speaking community. Increasing positive personal contact with people living with dementia may also improve social attitudes and decrease stigma (Phillipson et al, 2019). Employing bilingual HSCPs may increase cultural understanding of mainstream providers around the needs of Arabic-speaking Australians with dementia.

## Supplemental Material

Supplemental Material - Beliefs around help-Seeking and Support for Dementia in the Australian Arabic Speaking Community Click here for additional data file. Supplemental Material for Beliefs around help-Seeking and Support for Dementia in the Australian Arabic Speaking Community by Issra Allam, Meredith Gresham, Lyn Phillipson, Henry Brodaty, and Lee-Fay Low in Dementia: the international journal of social research and practice

## References

[bibr1-14713012231166170] Abbasi F. Paulsen E. (2019). Working with Muslim patients. American Psychiatric Association. https://psychiatry.org/psychiatrists/diversity/education/best-practice-highlights/working-with-muslim-patients

[bibr2-14713012231166170] Al Abed N. A. Davidson P. M. Hickman L. D. (2014). Healthcare needs of older Arab migrants: A systematic review. Journal of Clinical Nursing, 23(13–14), 1770–1784. 10.1111/jocn.12476 24329783

[bibr3-14713012231166170] Alhomaizi D. Alsaidi S. Moalie A. Muradwij N. Borba C. P. C. Lincoln A. K. (2018). An exploration of the help-seeking behaviors of Arab-Muslims in the US: A socio-ecological approach. Journal of Muslim Mental Health, 12(1), 19–48. 10.3998/jmmh.10381607.0012.102

[bibr4-14713012231166170] Alladi S. Hachinski V. (2018). World dementia: One approach does not fit all. Neurology, 91(6), 264–270. 10.1212/WNL.0000000000005941 29997191

[bibr5-14713012231166170] Alzheimer’s Disease International . (2019). World Alzheimer report 2019: Attitudes to dementia. https://www.alzint.org/resource/world-alzheimer-report-2019/

[bibr6-14713012231166170] Aryal R. (2017). Dealing with it myself: Supporting immigrant and refugee carers in Australia. Multicultural Centre for Women’s Health (MCWH). https://www.mcwh.com.au/wp-content/uploads/DWIMreport_A4_Book_v2_2018_OnlinePrint.pdf

[bibr7-14713012231166170] Atkin K. (2003). Ethnicity and the politics of the new genetics: Principles and engagement. Ethnicity and Health, 8(2), 91–109. 10.1080/13557850303561 14671764

[bibr8-14713012231166170] Australian Bureau of Statistics . (2015). Dementia and death in Australia (Cat. No. 3303.0). ABS.

[bibr9-14713012231166170] Australian Medical Association . (2020). AMA submission to the Royal Commission into aged care quality and safety – Response to consultation paper 1 - aged care program redesign: Services for the future. https://ama.com.au/system/tdf/documents/AMA_Submission_to_the_Royal_Commission_into_Aged_Care_Quality_and_Safety-aged_care_program_redesign.pdf?file=1&type=node&id=51843

[bibr10-14713012231166170] Bergeron C. D. Lagacé M. (2021). On the meaning of aging and ageism: Why culture matters. University of Toronto Quarterly, 90(2), 140–154. 10.3138/utq.90.2.06

[bibr11-14713012231166170] Bishop E. C. Shepherd M. L. (2011). Ethical reflections: Examining reflexivity through the narrative paradigm. Qualitative Health Research, 21(9), 1283–1294. 10.1177/1049732311405800 21508253

[bibr12-14713012231166170] Braun V. Clarke V. (2006). Using thematic analysis in psychology. Qualitative Research in Psychology, 3(2), 77–101. 10.1191/1478088706qp063oa

[bibr13-14713012231166170] Brijnath B. Gilbert A. S. Kent M. Ellis K. Browning C. Goeman D. Adams J. Antoniades J. (2021). Beyond crisis: Enacted sense-making among ethnic minority carers of people with dementia in Australia. Dementia, 20(6), 1910–1924. 10.1177/1471301220975641 33228396

[bibr14-14713012231166170] Colagiuri R. Thomas M. Buckley A. (2007). Preventing type 2 diabetes in culturally and linguistically diverse communities in NSW. Department of Health. https://www.diabetesaustralia.com.au/wp-content/uploads/Preventing-Type-2-Diabetes-in-Culturally-and-Linguistically-Diverse-Communities-in-NSW.pdf

[bibr15-14713012231166170] Connelly L. M. Peltzer J. N. (2016). Underdeveloped themes in qualitative research: Relationship with interviews and analysis. Clinical nurse specialist CNS, 30(1), 52–57. 10.1097/NUR.0000000000000173 26626748

[bibr16-14713012231166170] Daher-Nashif S. Hammad S. H. Kane T. Al-Wattary N. (2021). Islam and mental disorders of the older adults: Religious text, belief system and caregiving practices. Journal of religion and health, 60(3), 2051–2065. 10.1007/s10943-020-01094-5 33141404 PMC8137626

[bibr17-14713012231166170] El Masri A. Kolt G. S. Astell-Burt T. George E. S. (2017). Lifestyle behaviours of Lebanese-Australians: Cross-sectional findings from the 45 and up study. PloS ONE, 12(7). 10.1371/journal.pone.0181217 PMC5509310 28704508

[bibr18-14713012231166170] Etikan I. Musa S. A. Alkassim R. S. (2016). Comparison of convenience sampling and purposive sampling. American Journal of Theoretical and Applied Statistics, 5(1), 1–4. 10.11648/j.ajtas.20160501.11

[bibr19-14713012231166170] Godwill E. A. (2015). Fundamentals of research methodology: A holistic guide for research completion, management, validation and ethics. Nova Science Publishers Inc.

[bibr20-14713012231166170] Grossoehme D. Lipstein E. (2016). Analyzing longitudinal qualitative data: The application of trajectory and recurrent cross-sectional approaches. BMC Research Notes, 9(136). 10.1186/s13104-016-1954-1 PMC4776420 26936266

[bibr21-14713012231166170] Hamieh N. Sharara E. Salibi N. Mrad P. Chaaya M. (2019). Public knowledge of perceptions about and attitudes towards dementia: A cross-sectional survey among Lebanese primary health care attenders. Community Mental Health Journal, 55(8), 1362–1368. 10.1007/s10597-019-00436-2 31270647

[bibr22-14713012231166170] Harb C. (2016). The Arab region: Cultures, values, and identities. In Amer M. M. Awad G. H. (Eds.), Handbook of Arab American psychology (pp. 3–18). Taylor and Francis.

[bibr23-14713012231166170] Healthdirect . (2018). Dementia statistics. https://www.healthdirect.gov.au/dementia-statistics#:∼:text=More_than_50%25_of_residents,over_a_million_by_2058

[bibr24-14713012231166170] International Organization for Migration . (2020). World migration report 2020. https://publications.iom.int/books/world-migration-report-2020

[bibr25-14713012231166170] International Organization for Migration and League of Arab States . (2004). Arab migration in a Globalized world. https://publications.iom.int/system/files/pdf/arab_migration_globalized_world.pdf

[bibr26-14713012231166170] Jutlla K. (2013). Ethnicity and cultural diversity in dementia care: A review of the research. Journal of Dementia Care, 21(2), 33–39.

[bibr27-14713012231166170] Karam G. Itani L. (2013). Dementia : A review from the Arab region = الخرف : ملخص من العالم العربي. The Arab Journal of Psychiatry, 24(1), 77–84. 10.12816/0000102

[bibr28-14713012231166170] Kenny S. Mansouri F. Spratt P. (2005). Arabic communities and wellbeing: Supports and barriers to social connectedness. https://static1.squarespace.com/static/5b0fd5e6710699c630b269b1/t/5b41fc77f950b7059f903ab2/1531051135874/Arabic+Communities+and+Wellbeing.pdf

[bibr29-14713012231166170] Korstjens I. Moser A. (2018). Series: Practical guidance to qualitative research. Part 4: Trustworthiness and publishing. The European journal of general practice, 24(1), 120–124. 10.1080/13814788.2017.1375092 29202616 PMC8816392

[bibr30-14713012231166170] Livingston G. Huntley J. Sommerlad A. Ames D. Ballard C. Banerjee S. Brayne C. Burns A. Cohen-Mansfield J. Cooper C. Costafreda S. G. Dias A. Fox N. Gitlin L. N. Howard R. Kales H. C. Kivimaki M. Larson E. B. Ogunniyi A. Mukadam N. (2020). Dementia prevention, intervention, and care: 2020 report of the lancet commission. Lancet (London, England), 396(10248), 413–446. 10.1016/S0140-6736(20)30367-6 32738937 PMC7392084

[bibr31-14713012231166170] Mann C. J. (2003). Observational research methods. Research design II: Cohort, cross sectional, and case-control studies. Emergency medicine journal: EMJ, 20(1), 54–60. 10.1136/emj.20.1.54 12533370 PMC1726024

[bibr32-14713012231166170] Mazbouh-Moussa R. Ohtsuka K. (2017). Cultural competence in working with the Arab Australian community: A conceptual review and the experience of the Arab council Australia (ACA) gambling help counselling service. Asian Journal of Gambling Issues and Public Health, 7(1), 10–17. 10.1186/s40405-017-0029-0 29250480 PMC5725521

[bibr33-14713012231166170] Mukadam N. Cooper C. Livingston G. (2011). A systematic review of ethnicity and pathways to care in dementia. International Journal of Geriatric Psychiatry, 26(1), 12–20. 10.1002/gps.2484 21157846

[bibr34-14713012231166170] Nielsen T. R. Nielsen D. S. Waldemar G. (2021). Barriers in access to dementia care in minority ethnic groups in Denmark: A qualitative study. Aging and Mental Health, 25(8), 1424–1432. 10.1080/13607863.2020.1787336 32619352

[bibr35-14713012231166170] Phillips J. Pond C. D. Paterson N. E. Howell C. Shell A. Stocks N. P. Goode S. M. Marley J. (2012). Difficulties in disclosing the diagnosis of dementia: A qualitative study in general practice. The British journal of general practice: the journal of the Royal College of General Practitioners, 62(601), 546–553. 10.3399/bjgp12X653598 22867678 PMC3404332

[bibr36-14713012231166170] Phillipson L. Hall D. Cridland E. Fleming R. Brennan-Horley C. Guggisberg N. Frost D. Hasan H. (2019). Involvement of people with dementia in raising awareness and changing attitudes in a dementia friendly community pilot project. Dementia, 18(7–8), 2679–2694. 10.1177/1471301218754455 29363336

[bibr37-14713012231166170] Phillipson L. Hevink M. McAiney C. Gresham M. Maćkowiak M. Szczesniak D. Low L.-F. (2022). The utility of projective and enabling techniques to support engagement in research about dementia diagnosis and post-diagnostic support. University of Wollongong. [Manuscript submitted for publication].

[bibr38-14713012231166170] QSR International Pty Ltd (2020). NVivo (V12.6) https://www.qsrinternational.com/nvivo-qualitative-data-analysis-software/home

[bibr39-14713012231166170] Robotin M. C. Wallace J. Gallego G. George J. (2021). Hepatitis B and liver cancer: Community awareness, knowledge and beliefs of Middle Eastern migrants in Sydney, Australia. International Journal of Environmental Research and Public Health, 18(16), 8534. 10.3390/ijerph18168534 34444285 PMC8394558

[bibr40-14713012231166170] Sagbakken M. Spilker R. S. Nielsen T. R. (2018). Dementia and immigrant groups: A qualitative study of challenges related to identifying, assessing, and diagnosing dementia. BMC Health Services Research, 18(1), 910–924. 10.1186/s12913-018-3720-7 30497459 PMC6267848

[bibr41-14713012231166170] Saleh M. Barlow-Stewart K. Meiser B. Tucker K. Eisenbruch M. Kirk J. (2012). Knowledge, attitudes and beliefs of Arabic-Australians concerning cancer. Psycho-Oncology, 21(2), 195–202. 10.1002/pon.1884 22271540

[bibr42-14713012231166170] Saunders B. Sim J. Kingstone T. Baker S. Waterfield J. Bartlam B. Burroughs H. Jinks C. (2018). Saturation in qualitative research: Exploring its conceptualization and operationalization. Quality and quantity, 52(4), 1893–1907. 10.1007/s11135-017-0574-8 29937585 PMC5993836

[bibr43-14713012231166170] Setia M. S. (2016). Methodology series module 3: Cross-sectional studies. Indian Journal of Dermatology, 61(3), 261–264. 10.4103/0019-5154.182410 27293245 PMC4885177

[bibr44-14713012231166170] Shanley C. Boughtwood D. Adams J. Santalucia Y. Kyriazopoulos H. Pond D. Rowland J. (2012). A qualitative study into the use of formal services for dementia by carers from culturally and linguistically diverse (CALD) communities. BMC Health Services Research, 12(1), 354–354. 10.1186/1472-6963-12-354 23043332 PMC3523018

[bibr45-14713012231166170] Shanley C. Leone D. Santalucia Y. Adams J. Ferrerosa-Rojas J. E. Kourouche F. Gava S. Wu Y. (2013). Qualitative research on dementia in ethnically diverse communities: Fieldwork challenges and opportunities. American journal of Alzheimer's disease and other dementias, 28(3), 278–283. doi:10.1177/1533317513481099 10.1177/1533317513481099 PMC10852705 23512998

[bibr46-14713012231166170] Truswell D. (2016). The impact of dementia on migrant communities: A complex challenge in a globalised world. Alzheimer's, Dementia and Cognitive Neurology, 1. 10.15761/ADCN.1000102

[bibr47-14713012231166170] United Nations (2016). International migration Report 2015. Department of Economic and Social Affairs and Population Division. https://www.un.org/en/development/desa/population/migration/publications/migrationreport/docs/MigrationReport2015.pdf

[bibr49-14713012231166170] Zolezzi M. Alamri M. Shaar S. Rainkie D. (2018). Stigma associated with mental illness and its treatment in the Arab culture: A systematic review. The International journal of social psychiatry, 64(6), 597–609. 10.1177/0020764018789200 30019976

