# Supplementary material for: Beliefs around help-Seeking and Support for Dementia in the Australian Arabic Speaking Community
Source: Dementia (London). 2023 Mar 29;22(5):995–1009. doi: 10.1177/14713012231166170 (PMC10262330; doi:10.1177/14713012231166170)
Supplement: Supplemental Material - Beliefs around help-Seeking and Support for Dementia in the Australian Arabic Speaking Community [file sj-pdf-1-dem-10.1177_14713012231166170.pdf]

Appendix A: Stimulus materials used for projective technique interviewing for older people with dementia-like symptoms

Following introduction and rapport building with the interviewer, the following slides were used to structure discussion. Slides adapted from Phillipson et.al. 2022.

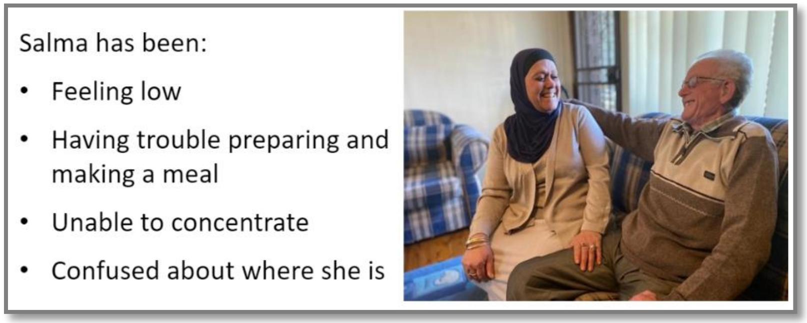

Fig. A.1. In this slide [illegible]
couple 'Salma' and '[illegible]
symptoms and asks, [illegible]
difficulty rememberi[illegible]
thoughts or feelings [illegible]

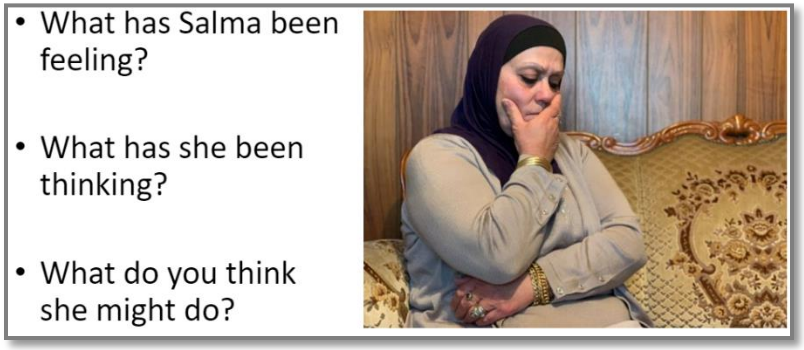

Fig. A.2. Participants [illegible]
thinking, how she is [illegible]
situation.

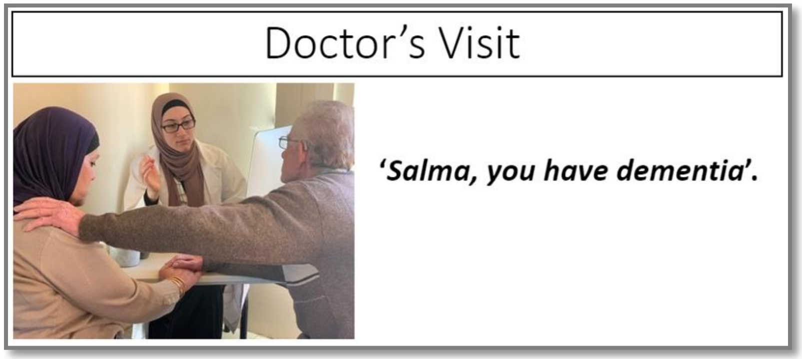

Fig. A.3. In this slide [illegible]
diagnosis of demen[illegible]
may be thinking an[illegible]
might be for the cou[illegible]

Appendix B: Stimulus materials used for projective technique interviewing for carers of older people

Following introduction and rapport building with the interviewer, the following slides were used to structure discussion. Slides adapted from Phillipson et.al. 2022.

Adel has noticed Salma is:

- Feeling low
- Has difficulty preparing and making a meal
- Unable to concentrate
- Confused about where she is

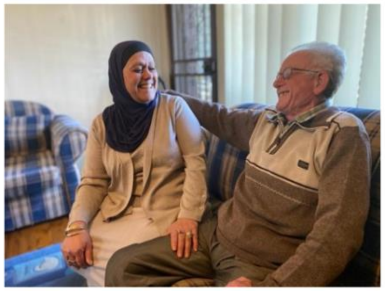

Fig. B.1. In this slide [illegible]
couple 'Salma' and [illegible]
symptoms and asks [illegible]
difficulty remembe[illegible]
thoughts or feelings [illegible]

- What has Adel been feeling?
- What has he been thinking?
- What do you think he might do?

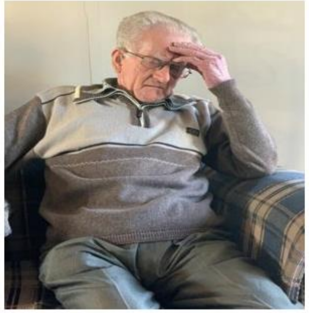

Fig. B.2. Carer parti[illegible]
be thinking, how he [illegible]
response to Salma' [illegible]

Doctor's Visit

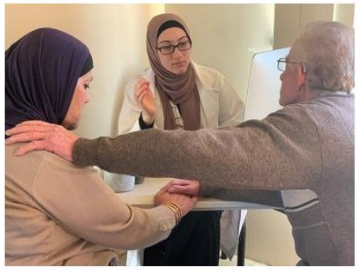

***'Salma, you have dementia'*.**

Fig.B.3. In this slide [illegible]
diagnosis of demen[illegible]
Adel may be thinki[illegible]
actions might be fo[illegible]

Appendix C: Slides used for projective technique interviewing with health or social care practitioners

Following introduction and rapport building with the interviewer, the following slides were used to structure discussion. Slides adapted from Phillipson et.al. 2022.

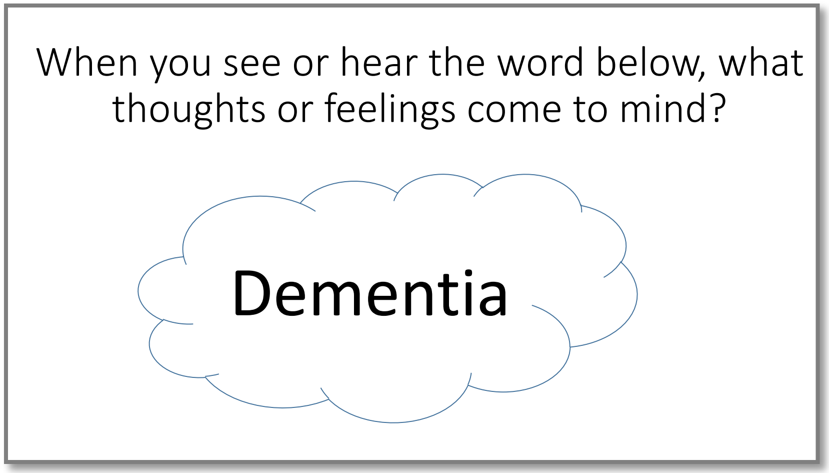

Fig. C.1. Word association projective technique for 'dementia'.

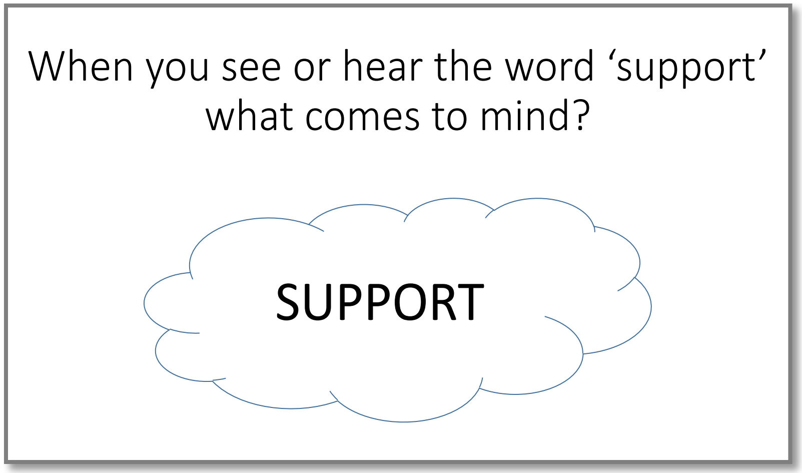

Fig. C.2. Word association projective technique for 'support'.
